# Strategies to maximise study retention and limit attrition bias in a prospective cohort study of men reporting a history of injecting drug use released from prison: the prison and transition health study

**DOI:** 10.1186/s12874-021-01380-0

**Published:** 2021-09-12

**Authors:** Ashleigh Cara Stewart, Reece Cossar, Shelley Walker, Anna Lee Wilkinson, Brendan Quinn, Paul Dietze, Rebecca Winter, Amy Kirwan, Michael Curtis, James R. P. Ogloff, Stuart Kinner, Campbell Aitken, Tony Butler, Emma Woods, Mark Stoové

**Affiliations:** 1 Behaviours and Health Risks, Burnet Institute, 85 Commercial Road, Melbourne, 3004 Australia; 2 School of Public Health and Preventive Medicine, Monash University, 85 Commercial Road, Melbourne, 3004 Australia; 3 Centre for Forensic Behavioural Science, Swinburne University of Technology and Forensicare, Melbourne, Australia; 4 National Drug Research Institute, Curtin University, Perth, Australia; 5 Department of Gastroenterology, St Vincent’s Hospital, Melbourne, Australia; 6 Monash Addiction Research Centre, Monash University, Melbourne, Australia; 7 Justice Health Unit, School of Population and Global Health, University of Melbourne, Melbourne, Australia; 8 Centre for Adolescent Health, Murdoch Children’s Research Institute, Melbourne, Australia; 9 Griffith Criminology Institute, Griffith University, Brisbane, Australia; 10 Mater Research Institute-UQ, University of Queensland, Brisbane, Australia; 11 School of Public Health and Community Medicine, University of Sydney, Sydney, Australia

**Keywords:** Attrition bias, People in prison, People who inject drugs, Cohort study

## Abstract

**Background:**

There are significant challenges associated with studies of people released from custodial settings, including loss to follow-up in the community. Interpretation of findings with consideration of differences between those followed up and those not followed up is critical in the development of evidence-informed policies and practices. We describe attrition bias in the Prison and Transition Health (PATH) prospective cohort study, and strategies employed to minimise attrition.

**Methods:**

PATH involves 400 men with a history of injecting drug use recruited from three prisons in Victoria, Australia. Four interviews were conducted: one pre-release (‘baseline’) and three interviews at approximately 3, 12, and 24 months post-release (‘follow-up’). We assessed differences in baseline characteristics between those retained and not retained in the study, reporting mean differences and 95% confidence intervals (95% CIs).

**Results:**

Most participants (85%) completed at least one follow-up interview and 162 (42%) completed all three follow-up interviews. Retained participants were younger than those lost to follow-up (mean diff − 3.1 years, 95% CI -5.3, − 0.9). There were no other statistically significant differences observed in baseline characteristics.

**Conclusion:**

The high proportion of participants retained in the PATH cohort study via comprehensive follow-up procedures, coupled with extensive record linkage to a range of administrative datasets, is a considerable strength of the study. Our findings highlight how strategic and comprehensive follow-up procedures, frequent contact with participants and secondary contacts, and established working relationships with the relevant government departments can improve study retention and potentially minimise attrition bias.

## Background

There has been a 20% rise in the global prison population since the year 2000, with more than 11 million people in prison worldwide on any given day [1]. Research has highlighted the disproportionate health, social, and economic disadvantage experienced by people in prison, including adverse childhood experiences [2–4], low educational attainment [5], high rates of unemployment and homelessness [6, 7], poor physical and mental health [8–11], substance use, dependence, and associated morbidities [12, 13], high rates of reimprisonment [14], and premature death [15]. Despite these outcomes being particularly acute for people in custodial settings with histories of injecting drug use (IDU), including two-year reimprisonment rates that exceed 80% [16], little is known about the post-release experiences of this population. This hinders the development of evidence-based community reintegration programs and the prevention of ongoing cycles of adverse health, social, and criminal justice outcomes in this group.

Longitudinal studies are essential for describing temporal associations between exposures and outcomes, and generate more reliable descriptions of causal pathways than cross-sectional studies [17]. Therefore, prospective cohort studies are particularly useful for informing policy and practice and identifying intervention opportunities. However, a key limitation of longitudinal studies is loss to follow-up (LTFU, or attrition), which can compromise the validity, reliability, and generalisability of study findings if attrition bias is present (i.e., if participants retained in the study are substantially different to those LTFU). There are significant challenges associated with following people after they are released from prison, and especially people who use/inject drugs. For example, transience, mental ill-health, and substance use can lead to frequently changing addresses and telephone numbers, and episodes of reimprisonment [18]. Describing effective methods that enhance retention of these groups in prospective follow-up can facilitate future research, and associated descriptions of attrition bias are critical to ensuring appropriate interpretation of findings and the development of evidence-based policies and practice. This study uses data from the Prison and Transition Health (PATH) prospective cohort study to (1) report on potential attrition bias in PATH and describe the baseline characteristics of those LTFU and those who completed at least one follow-up interview, and (2) describe the follow-up procedures we used to support high levels of post-release follow-up.

## Methods

### Study design

The Prison and Transition Health cohort study aims to characterise the transition from prison to the community setting among men in Victoria, Australia, who reported regular injecting drug use in the months prior to their imprisonment. The PATH study recruited 400 men in the few weeks preceding their release from one minimum, one medium, and one maximum-security prison in Victoria, Australia. The PATH study protocol, and baseline recruitment and participants’ characteristics, have been published elsewhere [19]. Study eligibility criteria included self-reporting IDU at least monthly in the 6 months prior to the index period of imprisonment (i.e., study recruitment prison episode), being aged ≥18 years at baseline, being sentenced (i.e., not on remand), and consenting to participate in the baseline and up to three interviews after release from prison. Year of index prison entry ranged from 2001 to 2016, with a median sentence length of 183 days (IQR 105–363). Baseline recruitment occurred between September 2014 and May 2016, a median of 39 days (IQR 15–69) prior to release from index imprisonment. Prior to baseline interviews, researchers spent approximately 15 min screening participants for eligibility, obtaining participants’ anticipated date of release, and scheduling interviews. Interviews were conducted as close to participants’ day of release as possible, recognising that cognisance of post-release circumstances was likely to increase as the day neared. Post-release follow-up interviews were scheduled for 3, 12, and 24 months post-release from participants’ index imprisonment episode. Follow-up interviews commenced in January 2015 and were completed in February 2019. These interviews were undertaken in the community or in prison (for participants reimprisoned, either sentenced or on remand, when due for their follow-up interview).

Participants also consented to have their interview data linked to a range of administrative health, social, and criminal justice datasets. The datasets and linkage processes available for the PATH cohort have been described previously [19]. All participants consented to data linkage during their baseline recruitment interview.

Participants were reimbursed AUD40 for their time and out-of-pocket expenses for participating in each follow-up interview completed in the community, in accordance with accepted practice [20]. Monetary reimbursement was not provided to participants in prison during baseline recruitment, or any subsequent follow-up interviews that were completed in prison, in accordance with Victorian Department of Justice and Community Safety (DJCS) policy.

### Follow-up strategies

Several strategies were adopted to reduce attrition. We collected comprehensive participant tracing information at baseline interviews in prison, an approach commonly used in cohort studies of marginalised “hard to reach” populations such as people who inject drugs, people living with hepatitis C, and those with a criminal justice history [21]. Participants were asked to provide primary contact information, including their full name, nicknames and any aliases, birthdates, Corrections Reference Numbers (unique identifying numbers assigned to people on remand or sentenced to prison by Corrections Victoria), and anticipated phone numbers, residential and postal addresses, and geographical locations after their release.

Participants were also invited to provide secondary contact information, including names and contact details of any individuals (e.g., family members, partners, friends) or services (e.g., health or social service providers, such as medication for opioid use disorder [MOUD] prescribers or dispensers, needle and syringe programs, crisis housing facilities, and Aboriginal health services) likely to know their whereabouts after their release. Researchers ensured that participants felt comfortable at the prospect of these individuals or services being contacted by the research team, explained how these details would be used for contacting them, and that information about the study would not be disclosed to secondary contacts (e.g., participants’ history of IDU). Once contacted, secondary contacts were asked to provide verbal consent to remain an ongoing point of contact, and if they did not consent were removed. The personal information of participants used for contacting them were stored in a secure, password-protected electronic database housed on a firewall-protected server in folders separate to primary survey data; these data were reviewed and updated throughout follow-up.

After participants were released, a range of strategies was implemented to enhance follow-up. An attempt was made to contact all participants in the initial days and weeks after release (prior to the first scheduled interview at three months post-release). The aim of this first contact was to confirm and/or update primary and secondary contact details of participants and, secondly, to remind them that they would be contacted for a follow-up interview three months after release.

Approximately a quarter of participants provided a personal phone number to contact them after release; fewer than half knew the address at which they would be living, and at least half provided no primary contact details. Thus, most participants were contacted via the secondary contact details (partners, family members, or friends) that around 90% of participants provided. Secondary contacts informed researchers of the whereabouts of participants (including if they had been reimprisoned, and where), and in many instances provided updated contact information allowing researchers to contact participants directly. Researchers posted letters to participants’ last known residential addresses (including secondary contacts), and several participants contacted the research team after receiving a letter. For some participants who remained uncontactable after these steps, letters were sent repeatedly unless we received them marked “return to sender”. Approximately two out of five participants provided details for a health service or social/welfare worker, which was intended as a mechanism for messages and letters to be provided to participants; a small number of participants were contacted via this method.

The Burnet Institute has a longstanding presence within community health services and local needle and syringe programs, and a consistent presence in known street-based drug markets for the purposes of research with community cohorts of people who use/inject drugs throughout Melbourne, Victoria [22]. This enabled some participants to be contacted opportunistically in the field.

In light of known high rates of reimprisonment in this population [14], we established a system to contact participants who may have been reimprisoned. Following advice from secondary contacts that a participant had returned to custody, or numerous unsuccessful attempts (over several months) to contact participants at each scheduled follow-up, a list of participant names was submitted to the DJCS for review. For those who were reimprisoned in Victorian prisons, DJCS indicated the facilities where participants were located, which enabled researchers to complete follow-up interviews. Of 748 follow-up interviews in PATH, 206 (28%) were conducted in prison.

During follow-up, the research team adopted other strategies to contact participants via social media, primarily Facebook. Generic messages that did not disclose any personal information or information about the study were sent via the direct message platform, when attempts to make contact via phone and letters were unsuccessful; this approach proved successful for some participants.

### Statistical analysis

Follow-up rates were calculated for each follow-up interview. Participants who died prior to their interview due date, as determined through probabilistic linkage with Australia’s National Death Index (NDI – using first and last names, gender, date of birth, and last known residential address), were excluded from the denominator in calculation of follow-up rates for that and any subsequent interviews. Follow-up rates are calculated as: (1) per study protocol, referring to follow-up as scheduled at 3, 12, and 24 months after release from participants’ index imprisonment episode; and (2) follow-up waves, referring to the sequence of first, second, and third interview; for example, if a participant did not complete their scheduled 3-month interview but then completed their 12-month interview, this interview would be their first follow-up. Follow-up rates are illustrated graphically, and because data were skewed, the median and interquartile ranges (IQR) are presented for the number of months since index prison release to when follow-up interviews occurred.

Attrition bias was assessed by comparing differences in baseline characteristics between participants who completed at least one follow-up interview (hereafter referred to as “participants retained”) and participants who were LTFU after baseline interview (hereafter referred to as “participants LTFU”).

Variables pertinent to analysing the association of key outcomes and exposures that address the aims of the PATH study were selected from the sociodemographic, adverse childhood experiences, substance use, and criminal justice domains; these included age at baseline (years), being born outside of Australia (no, yes), identifying as Aboriginal and Torres Strait Islander (no, yes), in a stable relationship prior to index imprisonment, including married and de facto (no, yes), number of years of education completed (< 10, ≥10 years), main income source prior to index imprisonment (government pension, illegal activities, paid employment, other sources), accommodation prior to index imprisonment (private rental, family home, public housing, no fixed address, owner occupied), ever removed from family as a child (no, yes), ever declared a ward of the state (no, yes), parent or caregiver to a child (no, yes), child protection involvement with care of children (among parents/caregivers; no, yes), mental illness diagnosis ever (no, yes), history of suicide attempt (no, yes), age of first drug injection (years), duration of IDU (calculated as the difference between current age and reported age of first drug injection; years), total drug injections in the week prior to imprisonment (count), ever used a syringe after someone else (no, yes), ever injected drugs during an episode of imprisonment (no, yes), hepatitis C virus antibody dry blood spot test (DBS) result collected at baseline interview (negative, positive), history of drug overdose (no, yes), receiving MOUD at time of baseline interview (no, yes), history of juvenile detention (no, yes), and number of adult imprisonment episodes at baseline (count).

We generated descriptive statistics for participant characteristics, stratified by those who were and were not retained in the study (no, yes). To compare characteristics between participants retained in the study and those LTFU, we checked the underlying distribution of the data and for normally distributed continuous variables we reported mean differences and 95% confidence intervals (95% CIs), and for categorical variables we reported mean differences in proportions and 95% CIs. All analyses were conducted using Stata 15.1 for Windows [23].

## Results

**Fig. 1:**
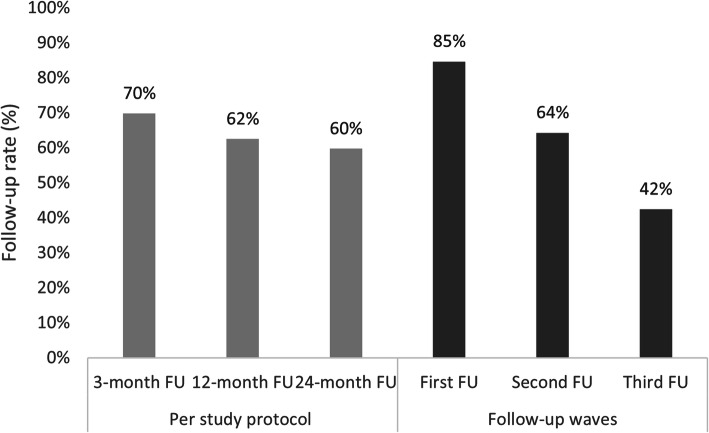
Follow-up rates at 3, 12 and 24-months per study protocol (*n =* 336) and by first, second and third follow-up waves (*n =* 336)

Of the 400 men recruited into the PATH study, five died prior to their three-month interview due date. Of the remaining 395 men, 336 (85%) completed at least one follow-up interview (64 participants were LTFU after baseline interview). During the 24-month observation period, 18 participants died. Of those eligible at each follow-up interview, as per study protocol, 70% (*n* = 277/395) completed a 3-month follow-up, 62% (*n* = 243/389) completed a 12-month follow-up, 60% (*n* = 228/382) completed a 24-month follow-up, and 42% (*n* = 162/382) completed all three follow-up interviews (Fig. 1). Three-month follow-up interviews occurred a median of 3.6 months (IQR 3.0–4.9 months), 12-month follow-up occurred a median of 13.1 months (IQR 12.0–15.8 months), and 24-month follow-up interviews occurred a median of 26.1 months (IQR 24.3–30.3 months) post-release from index prison episode.

Follow-up rates by wave were 85% (*n* = 336/395) at first follow-up, 64% (*n* = 250/389) at second follow-up, and 42% (*n* = 162/382) at third follow-up (Fig. 1). First follow-ups occurred a median of 4.0 months (IQR 3.1–7.2 months), second follow-ups occurred a median of 14.5 months (IQR 12.3–19.6 months), and third follow-ups occurred a median of 25.8 months (IQR 24.3–29.0 months) post-release from index prison episode.

The mean age of retained participants at baseline was 35.3 years (SD 8.0). Seventeen per cent identified as Aboriginal and/or Torres Strait Islander, 91% were born in Australia, and 41% had completed less than 10 years of education (other sample characteristics are detailed in Table 1). Participants retained were on average younger than those LTFU (mean diff − 3.11, 95% CI -5.33, − 0.88). There were no other differences observed in baseline characteristics between those retained and those LTFU (Table 1).

**Table 1:** Baseline characteristics among participants retained and participants LTFU from baseline interview in the PATH study (*N* = 400)

	Retained (*n* = 336)	Lost to follow-up (*n* = 64)	Diff. in props^d^/ mean diff.^e^	95% CI
Age, years - mean (SD)^e^	35.3 (8.0)	38.5 (9.5)	−3.11	−5.33, − 0.88
Recruitment prison security level – n (%)				
Low	86 (26)	22 (34)	−0.09	− 0.21, 0.04
Medium	95 (28)	16 (25)	0.03	−0.08, 0.15
Maximum	155 (46)	26 (41)	0.06	−0.08, 0.19
Born outside Australian (vs. in Australia) – n (%)	304 (91)	54 (84)	− 0.06	−0.15, 0.03
Aboriginal and Torres Strait Islander (vs. no) – n (%)	56 (17)	10 (16)	0.01	−0.09, 0.11
Stable relationship prior to prison (vs. no) – n (%)	123 (37)	24 (38)	−0.01	−0.14, 0.12
Education < 10 years (vs. ≥10 yrs.) – n (%)	136 (41)	31 (48)	− 0.08	− 0.21, 0.05
Main income source before prison – n (%)				
Government payment^a^	163 (49)	35 (55)	−0.07	−0.20, 0.07
Illegal activities	132 (39)	21 (33)	0.06	−0.06, 0.19
Paid work (inc. cash in hand)	31 (9)	5 (8)	0.01	−0.06, 0.09
Other sources	8 (2)	2 (3)	−0.01	−0.05, 0.04
Accommodation prior to incarceration – n (%)				
Private rental (single or shared)	90 (27)	18 (28)	−0.00	−0.12, 0.12
Family home	66 (20)	11 (17)	0.03	−0.07, 0.14
Public housing	60 (18)	16 (25)	−0.07	− 0.18, 0.05
No fixed address^b^	82 (24)	15 (23)	0.02	−0.09, 0.13
Owner occupied	20 (6)	3 (5)	0.02	−0.04, 0.07
Removed from parent’s care, ever (vs no) – n (%)	86 (26)	14 (22)	0.03	−0.08, 0.15
Declared state ward, ever (vs no) – n (%)	50 (16)	12 (19)	−0.04	−0.14, 0.07
Parent/caregiver (vs no) – n (%)	218 (65)	49 (77)	−0.11	−0.22, 0.01
Child protection involvement with children^c^ (vs no) – n (%)	98 (29)	20 (31)	−0.02	−0.14, 0.10
Mental illness diagnosis, ever (vs no) – n (%)	272 (81)	52 (81)	−0.00	−0.11, 0.10
Suicide attempt, ever (vs no) – n (%)	159 (47)	23 (36)	0.11	−0.02, 0.24
Age of first injection, years – mean (SD)^e^	18.4 (5.4)	19.2 (6.8)	−0.81	−2.33, 0.71
Duration of IDU, years – mean (SD)^e^	17 (8.8)	19 (10.1)	−2.30	−4.71, 0.12
Total injections week prior to prison – mean (SD)^e^	29 (25.3)	33 (25.8)	−3.61	−10.36, 3.14
Shared used syringe, ever (vs no) – n (%)	229 (68)	46 (72)	−0.04	−0.16, 0.08
Injected in prison, ever (vs no) – n (%)	159 (47)	30 (47)	0.00	−0.13, 0.14
HCV antibody DBS positive (vs negative) – n (%)	273 (81)	55 (86)	−0.05	−0.14, 0.05
Overdose, ever (vs no) – n (%)	198 (59)	32 (50)	0.09	−0.04, 0.22
MOUD, at baseline (vs no) – n (%)	139 (41)	26 (41)	0.01	−0.12, 0.14
Juvenile incarceration (vs no) – n (%)	150 (45)	25 (39)	0.05	−0.09, 0.18
Adult imprisonment episodes, mean (SD)^e^	4 (3.9)	4 (3.1)	0.66	−0.36, 1.68

*MOUD* medication for opioid use disorder, *HCV* hepatitis C virus, *DBS* dry blood spot test

^a^Includes Newstart allowance, disability support pension, parent allowance, and carer allowance

^b^Includes residing in a boarding house/hostel, crisis accommodation, transitional housing, couch surfing, staying in a squat, sleeping rough

^c^Among those reporting to be parents/caregivers

^d^mean differences in proportions for categorical variables

^e^mean difference for continuous variables

## Discussion

This study demonstrates the success of the range of measures implemented to enhance retention in post-release interviews for a cohort study of people with a history of IDU recruited in prison. Minimal attrition bias in the PATH study supports the validity of study findings based on behavioural data collected from the cohort to describe participants’ trajectories over two years post-release. Apart from age, there were no significant differences in baseline characteristics between participants retained and participants LTFU; this contrasts with population cohort studies in which attrition is often differential [24]. The collection of detailed contact tracing information, particularly secondary contacts, and comprehensive approaches to enhance follow-up via experienced fieldworkers resulted in a high study retention rate compared to other studies involving similarly marginalised and transient target populations, including people who inject drugs [22, 25] and people released from prison [18].

A strength of the PATH study design is the duration of the behavioural observation period. Other longitudinal studies of criminal justice-involved populations and people who inject drugs have been limited by short durations of follow-up [26, 27], or retrospective study designs [28, 29]. The duration of the behavioural observation period allows for a greater understanding of the longer-term health, social, and criminal justice trajectories of this population, and assists in ascertaining health-related needs and identifying crucial intervention opportunities to improve health and social outcomes for this population. Complementary to the extensive behavioural data is the comprehensive retrospective and prospective data linkage to a range of administrative health, social, and criminal justice datasets; to which all participants consented to during their baseline recruitment interview. This is a particular strength of the PATH study design, allowing for the validation of self-report data and measurement of morbidity and mortality among marginalised sub-populations [30], assessment of service utilisation and other health and social outcomes among participants LTFU, and understanding of attrition in cohort studies (e.g., by using linked data from the NDI to exclude deceased participants from follow-up rate calculations). Participant follow-up is highly resource intensive [31], so data linkage is useful in analysing longer-term trajectories well beyond the period of direct participant follow-up (linkage to administrative data for PATH is planned for 2, 5 and 10 years after index release).

There are inherent challenges associated with the prospective follow-up of people released from custody, including maintaining ongoing contact, due to high rates of transience and episodes of reimprisonment. Several challenges were encountered in the follow-up of participants in the PATH study; our responses may prove informative and beneficial for the design of future research with highly marginalised populations, including those in contact with the justice system and/or engaging in stigmatised behaviours, such as IDU. Of particular utility was the ability to complete interviews in prison. This not only supported the high study retention, but allowed for an examination of experiences and factors associated with recidivism and reimprisonment (a core aim of the PATH study) and experiences of transitioning out of prison beyond the baseline index imprisonment episode. However, there were also major obstacles to follow-up interviews in prison. Prisons are dynamic environments with frequent transfers of individuals, access constraints due to prison operations, industrial action, lack of custodial staff to facilitate access to participants, and rapid releases back into the community, especially for those on remand. These and other factors unique to the prison environment disrupted interview scheduling and caused frequent cancellations. While many reimprisoned participants were able to be interviewed, some were either released or transferred prior to contact from the study team. This resulted in interviews being missed, or interviews only being possible via further liaison with DJCS, highlighting the urgency of participant follow-up in prison. Moreover, Australian correctional systems are organised at the state/territory level, and as such, we were unable to identify interstate imprisonment, which would have required complex arrangements with multiple correctional authorities.

Collecting comprehensive details for participants’ nominated secondary contacts was crucial to maintaining contact during the study, particularly given the paucity of primary contact information available. The development and implementation of a protocol to contact participants via publicly available social media information proved successful in maintaining current contact details and contacting participants LTFU, and should be considered for future cohort studies. Although not as successful as other methods, the collection of contact details for participants’ health and social service providers allowed messages and letters to be passed on to participants, which for some was a useful mechanism for follow-up. Moreover, Burnet Institute’s established relationships with, and presence within, community services and extensive experience working with people who use/inject drugs, including staff trained in field-based data collection and questionnaire administration [22], allowed for some participants to be contacted opportunistically.

The high follow-up rate in the PATH study can be attributed to the combination of follow-up strategies we employed. As such, researchers should employ as many strategies as possible when following up participants, with repeated phone contact indicated as the most useful follow-up strategy [31], although this requires substantial time, effort, and cost. Finally, the data collected in the study was not sufficient to determine the relative efficacy of follow-up strategies. To inform the development of follow-up protocols for cohort studies and improve study retention, future studies should consider collecting data on the degree of success associated with specific strategies used for participant follow-up.

## Conclusion

Attrition bias is a limitation inherent to longitudinal studies and is especially prominent in studies of people involved in the criminal justice system. The high proportion of participants retained in the PATH cohort is a strength of this study. Strategic and comprehensive follow-up procedures, frequent contact with participants and secondary contacts, and established working relationships with relevant government departments can facilitate study retention and minimise attrition bias. Behavioural data collected as part of the PATH study, coupled with data linkage with various administrative datasets, will strengthen the validity of the study outcomes and their translation into policy and practice.
